# Identification of Potential Inhibitors for the Treatment of Alkaptonuria Using an Integrated In Silico Computational Strategy

**DOI:** 10.3390/molecules28062623

**Published:** 2023-03-14

**Authors:** Sumera Zaib, Nehal Rana, Nadia Hussain, Hanan A. Ogaly, Ayed A. Dera, Imtiaz Khan

**Affiliations:** 1 Department of Basic and Applied Chemistry, Faculty of Science and Technology, University of Central Punjab, Lahore 54590, Pakistan; 2 Department of Pharmaceutical Sciences, College of Pharmacy, Al Ain University, Al Ain P.O. Box 64141, United Arab Emirates; 3 AAU Health and Biomedical Research Center, Al Ain University, Abu Dhabi P.O. Box 144534, United Arab Emirates; 4 Chemistry Department, College of Science, King Khalid University, Abha 61421, Saudi Arabia; 5 Biochemistry and Molecular Biology Department, Faculty of Veterinary Medicine, Cairo University, Giza 12211, Egypt; 6 Department of Clinical Laboratory Sciences, College of Applied Medical Sciences, King Khalid University, Abha 62529, Saudi Arabia; 7 Manchester Institute of Biotechnology, The University of Manchester, 131 Princess Street, Manchester M1 7DN, UK

**Keywords:** alkaptonuria, nitisinone, homogentisate dioxygenase, 4-hydroxyphenylpyruvate dioxygenase, ReCore

## Abstract

Alkaptonuria (AKU) is a rare genetic autosomal recessive disorder characterized by elevated serum levels of homogentisic acid (HGA). In this disease, tyrosine metabolism is interrupted because of the alterations in homogentisate dioxygenase (HGD) gene. The patient suffers from ochronosis, fractures, and tendon ruptures. To date, no medicine has been approved for the treatment of AKU. However, physiotherapy and strong painkillers are administered to help mitigate the condition. Recently, nitisinone, an FDA-approved drug for type 1 tyrosinemia, has been given to AKU patients in some countries and has shown encouraging results in reducing the disease progression. However, this drug is not the targeted treatment for AKU, and causes keratopathy. Therefore, the foremost aim of this study is the identification of potent and druggable inhibitors of AKU with no or minimal side effects by targeting 4-hydroxyphenylpyruvate dioxygenase. To achieve our goal, we have performed computational modelling using BioSolveIT suit. The library of ligands for molecular docking was acquired by fragment replacement of reference molecules by ReCore. Subsequently, the hits were screened on the basis of estimated affinities, and their pharmacokinetic properties were evaluated using SwissADME. Afterward, the interactions between target and ligands were investigated using Discovery Studio. Ultimately, compounds **c** and **f** were identified as potent inhibitors of 4-hydroxyphenylpyruvate dioxygenase.

## 1. Introduction

**Figure 1:**
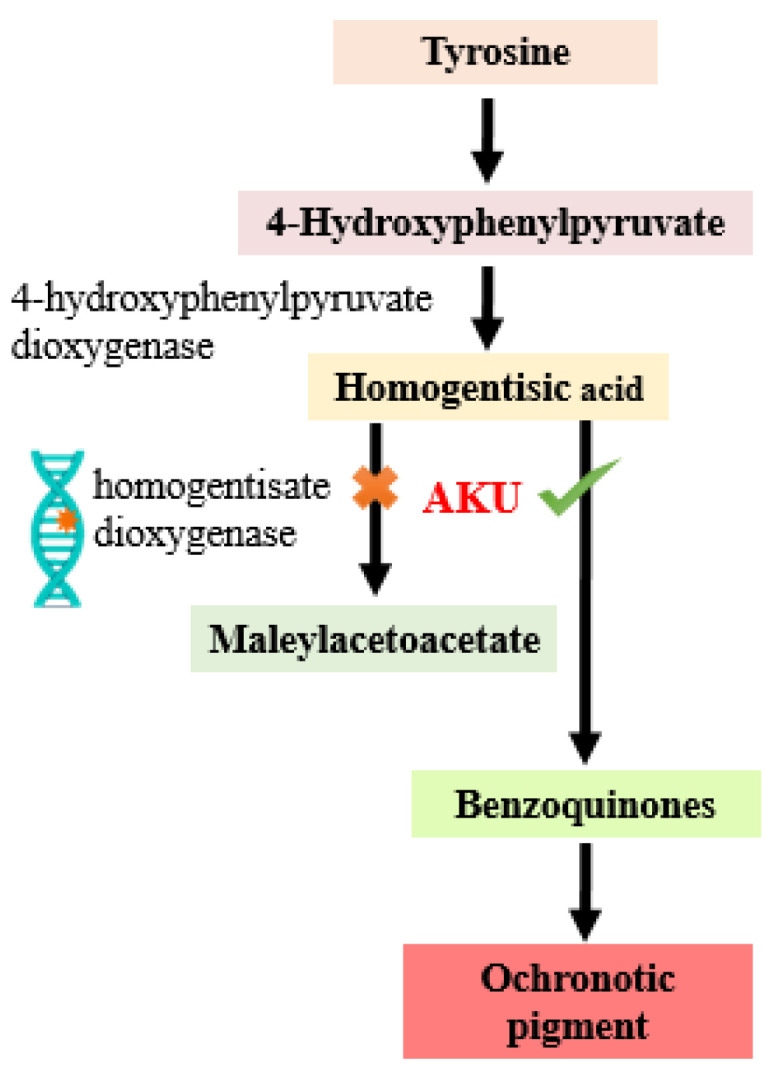
Metabolic error in Alkaptonuria (AKU). In AKU, the gene encoding homogentisate dioxygenase is mutated thus converting homogentisic acid to benzoquinones and producing bluish-black pigment in connective tissues, also known as ochronotic pigment.

Alkaptonuria (AKU) is a rare monogenic hereditary multisystemic disease inherited in an autosomal-recessive pattern [1]. The disease is known since 1500 BC as several reports have suggested AKU symptoms in Egyptian mummies of that time [2]. It is ultra-rare as its incidence is 1:250,000 to 1,000,000 with most reported cases in India, Slovakia, and Jordan due to consanguineous marriages [3]. It was first described by Dr. Archibald Garrod who described it as genetic disease and coined the concept of “inborn errors of metabolism” [4]. Furthermore, Boedeker suggested the name of AKU which was derived from the term Alkapton. The term Alkapton is of Greek origin that is kápton which means “to gulp down” [5]. It is characterized by high levels of serum and urine homogentisic acid (HGA) due to mutations in the nucleotide sequence of homogentisate 1,2-dioxygenase (HGD) [1]. These alterations in the nucleotide sequence interfere with functioning of HGD [6]. HGD metabolizes HGA, an intermediate product of phenylalanine/tyrosine pathway which is converted to fumarate and acetoacetate in normal individuals but not in AKU as shown in Figure 1 [7,8].

HGA being a reducing agent is oxidized by a slow spontaneous irreversible process imparting black color to the urine and was first explained as “as black as ink” by Scribonius in 1584 [9,10]. In addition to the blackening of urine, the eyes and ears also develop pigmentation, but it takes two to three decades to be visible externally [10]. The pigment is actually a bluish-black melanin-like pigment formed by the oxidation of HGA in connective tissues by a process known as ochronosis [3]. This melanin-like pigment is actually benzoquinone acetic acid [11]. Moreover, the patients also suffer from knee and backbone pain that originates gradually in two to three decades [12]. Additional manifestations of AKU include arthritis, aortic stenosis, joint damage, musculoskeletal tears, renal stones, and calcification of the vascular system [8,13,14,15]. The destruction of joints in AKU is attributed to the alterations in extracellular matrix, deterioration of collagen fibers and loss of proteoglycans [2,16]. Consequently, the cartilage becomes stiff and unable to bear the mechanical stress. This whole process begins in calcified tissues and continues to the hyaline cartilage inducing remodeling and destruction of subchondral plate [17]. It is not understood how HGA interacts with collagen matrix at the molecular level. A microscopic analysis of pigmented cartilage revealed a close relationship between early pigmentation and collagen fiber periodicity [10]. Although collagen fibers abide by the pigmentation, but biomechanical and metabolic alterations, such as those that take place in cartilage as a result of ageing naturally, make tissues vulnerable to ochronosis [10]. After the depletion of protective components such as proteoglycans and glycosaminoglycans, HGA easily binds with collagen [18].

In case of bone remodeling, two factors namely RANKL/osteoprotegerin (OPG) ratio and the Wnt/*β*-catenin signaling pathway are vital. RANKL/osteoprotegerin (OPG) ratio has significant role in the formation of osteoclast; on the other hand, Wnt/*β*-catenin signaling pathway controls the division and differentiation of osteoblasts [19]. In AKU, elevated levels of HGA inhibits Wnt signaling pathway by induced deterioration of osteoblast functionality [20].

Cardiac symptoms linked to alkaptonuria are minimal. The most common is aortic ochronosis that causes aortic stenosis and requires surgical treatment. The pulmonary and tricuspid valves are the subsequent rarely afflicted, followed by the mitral valve [21]. Certain patients have indeed reported involvement of the aortic, sternal, and coronary arteries. Despite the fact that alkaptonuric patients’ aortic walls have pigmentation, there is evidence of reduced aortic distensibility [21]. Although it is rare, but some AKU patients can also manifest neurological symptoms such as Parkinsonism. Basically, HGA fuse with the melanin to form a complex in substantia nigra which interferes with the formation of dopamine-melanin complex [22]. Patients may also suffer from depression due to physical impairment from pigment buildup in the skin, darkened urine, acute arthralgia and rigidity, and recurrent surgeries [22]. The musculoskeletal abnormalities in AKU are often deferred till the fourth decade of life and frequently precedes with the onset of spinal arthropathy [23].

Recently, AKU has been categorized as secondary amyloidosis because of the deposition of serum amyloid A (SAA) fibers. The SAA is a biomarker of inflammation as produced at 100–1000 times in higher amounts than normal plasma levels (4–6 mg/L) during chronic inflammation [24]. SAA fibers are mainly produced by the under expression of cathepsin D in the chondrocytes in AKU patients which has role in regulating the levels of secondary amyloid fibrils [25]. In addition to high levels of SAA fibers, HGA can also disrupt the Hedgehog signaling in chondrocytes by shortening the cilia and upregulating the expression of Gli-1 protein [26].

The disease can be diagnosed by measuring the excretory level of HGA in urine which is estimated to be 8 g/day [27]. However, the normal urine levels of HGA in healthy individuals are <1.1 µmol/L as compared to AKU patients in which millimolar levels of HGA exists [7,16]. This HGA secreted in the urine is not only obtained from the glomerular filtration but a major percentage of HGA urine is obtained from the renal tubular secretion [28]. In addition to HGA in urine, the bacterial load of urine of infected male patients is also 2–3 times greater than female AKU patients [29]. Moreover, it can be confirmed by the genetic analysis for mutations in HGD gene. Three mutations namely M368V, V300G and P230S are most common in the HGD gene in AKU patients [6]. The ocular symptoms are also beneficial in the diagnosis of AKU as 83% of the AKU patient’s manifest symmetric scleral pigmentation [30]. This pigmentation is due to the deposition of pigment specifically in the sclera (Osler’s sign) having impairment in ocular tendons [30,31].

AKU can also be misdiagnosed due to its rarity and asymptomatic presentation for several years. The impairment and ochronosis of joints can be confused with the rheumatoid arthritis, hyperparathyroidism and ankylosing spondylitis [32], whereas ocular symptoms can be mistakenly diagnosed as ocular melanosarcoma [33].

To date, alkaptonuria is without a specific cure; however, it is being managed via physiotherapy, painkillers, and surgery for replacing joints [34]. Additionally, several studies have reported the use of nitisinone, which is a US Food and Drug Administration (FDA)-approved drug for tyrosinemia type 1, to ameliorate AKU by arresting ochronosis [35]. However, the FDA has not approved its use for alkaptonuria treatment because the use of nitisinone has caused acquired tyrosinosis, elevated liver transaminases, and corneal crystals [1,27,36]. Therefore, alkaptonuria specific drug is required with minimal side effects to treat the patients. Therefore, in the current study, computational approaches were used to identify potent and druggable inhibitors of alkaptonuria through targeting 4-hydroxyphenyl pyruvate dioxygenase. This enzyme is an alpha-keto acid dependent oxygenases and catalyzes the oxidative decarboxylation of 4-hydroxyphenyl pyruvate (HPP) to form homogentisic acid (HGA) [37]. The acquired results showed promising compounds that could further be validated through experimental work and serve as a potential treatment for the very rare inherited disorder, alkaptonuria.

## 2. Results and Discussion

### 2.1. Target Identification

**Figure 2:**
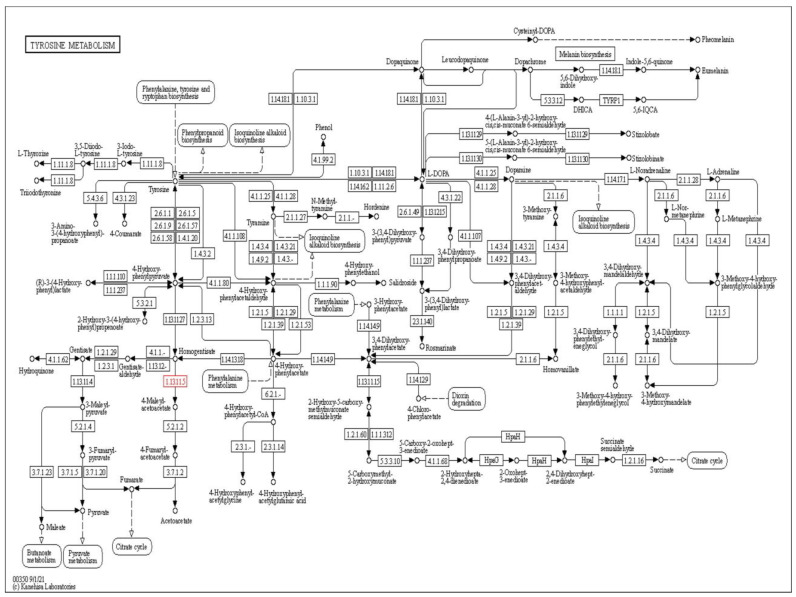
Tyrosine metabolism pathway by KEGG. The highlighted enzyme commission number (EC number) is representing the mutated enzyme in alkaptonuria.

According to the literature and KEGG pathway, homogentisate 1,2-dioxygenase (EC number: 1.13.11.5) was mutated, resulting in the enhanced level of homogentisic acid, as shown in Figure 2. 4-Hydroxyphenylpyruvate dioxygenase was the suitable target for treating AKU due to its role in the synthesis of homogentisic acid. The targeted protein was downloaded from the PDB database (PDB id: 3ISQ). This protein is made up of just one chain A having 393 amino acids, and so far no mutations have been reported in this protein. In addition, its resolution was 1.75 Å [38].

### 2.2. Binding Site Prediction

**Figure 3:**
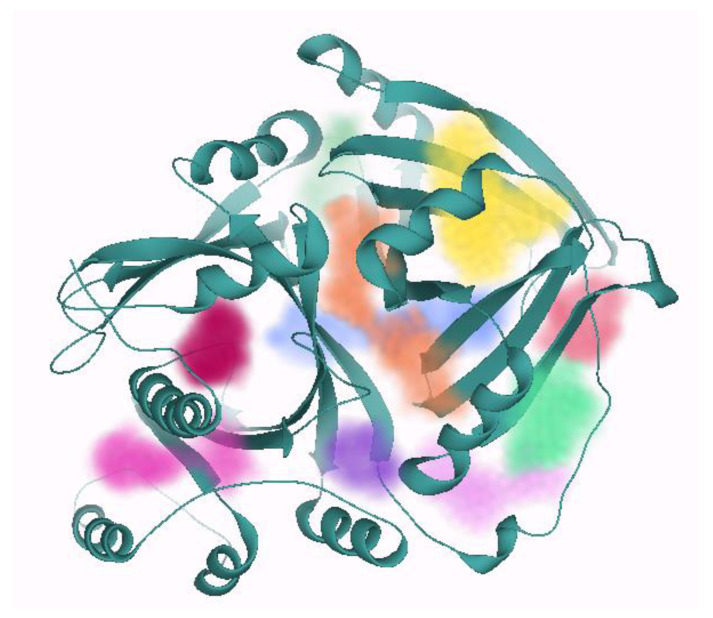
A visualization of all the unoccupied binding sites in 4-hydroxyphenylpyruvate dioxygenase. It consists of a total of 10 binding pockets represented by different colors, but the one represented in yellow is selected for having the highest DoGSiteScore. The color of each unoccupied site is numbered and described in Table 1.

The protein was loaded in SeeSAR protein mode, followed by the selection of a co-crystalline ligand with the most binding affinity than the other four natural ligands in order to automatically select the binding site for docking. However, the binding site just consisted of a limited number of amino acid residues that were expanded by shifting the protein in the binding site mode. The binding site mode enables the visualization of all the unoccupied binding pockets in the target protein as represented in Figure 3. It consists of 10 binding pockets with different acceptors, donors, hydrophobicity, DoGSiteScore, surface area, and volume, as shown in Table 1. The one in yellow was selected because it has the highest DoGSiteScore that accurately predicts the druggable pocket in a protein [39]. Moreover, DoGSiteScore was preferred for the selection of binding site because it has been shown to outperform other existing methods for druggable pocket prediction. The DoGSiteScore is predicted by utilizing a machine learning algorithm that considers various physical and chemical parameters, such as solvent accessibility, flexibility, and amino acid composition to predict the potential binding sites for small molecules in a protein [39].

### 2.3. Ligand Evaluation

**Figure 4:**
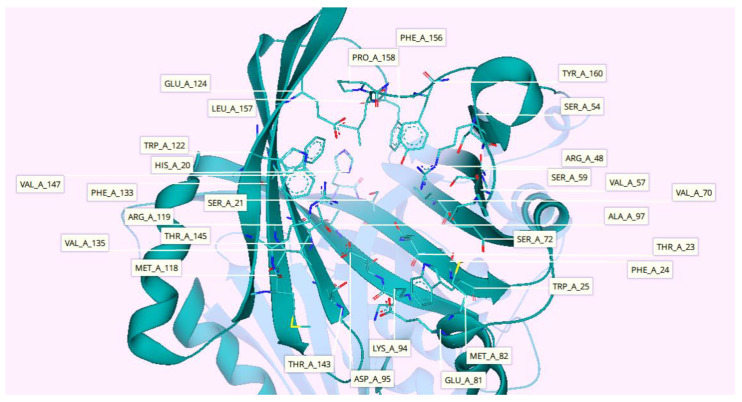
Three-dimensional (3D) structure of 4-hydroxyphenylpyruvate dioxygenase obtained from PDB. The figure highlights the active pocket residues (pale teal) in stick conformation, while other protein residues are represented in cartoon conformation (dark teal). Active site constitutes a total of 29 residues, including His20, Ser21, Thr23, Phe24, Trp25, Arg48, Ser54, Val57, Ser59, Val70, Ser72, Glu81, Met82, Lys94, Asp95, Ala97, Met118, Arg119, Trp122, Glu124, Phe133, Val135, Thr143, Thr145, Val147, Phe156, Leu157, Pro158, and Tyr160.

As reported in the literature, nitisinone is being used in some countries to treat AKU, but it is not a specific treatment to this disease. Therefore, nitisinone was docked with the 4-hydroxyphenylpyruvate dioxygenase in the docking mode of SeeSAR. The target-nitisinone complex was transported to the binding site mode where surrounding residues were added to the selected pocket to enhance the interaction sites. Therefore, total active site residues were increased from 23 to 29 as elucidated in Figure 4.

**Figure 5:**
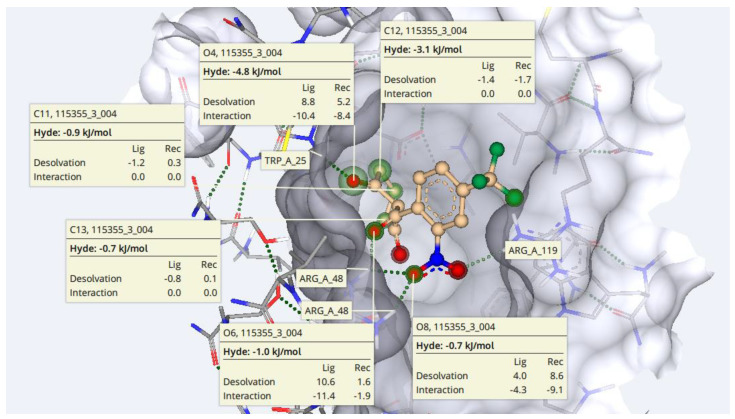
HYDE estimation of nitisinone. According to HYDE calculations, O4, O6, O8, C11, C12, and C13 have a major contribution to the overall estimated affinity of the docked complex.

Furthermore, the HYDE scoring of each atom in the nitisinone was determined in order to estimate the contribution of the sole atom in the overall millimolar estimated affinity of nitisinone. It can be seen in Figure 5 that oxygen atoms at position number 4, 6, and 8 have HYDE energy of −4.8, −1.0, and −0.7 kJ/mol, respectively. Similarly, the HYDE of carbon atoms at position 11, 12, and 14 are −0.9, −3.1, and −0.7 kJ/mol, respectively. The lower the HYDE scoring, the higher will be the importance of that particular atom in the overall structure [40].

### 2.4. ReCore and Molecular Docking

**Figure 6:**
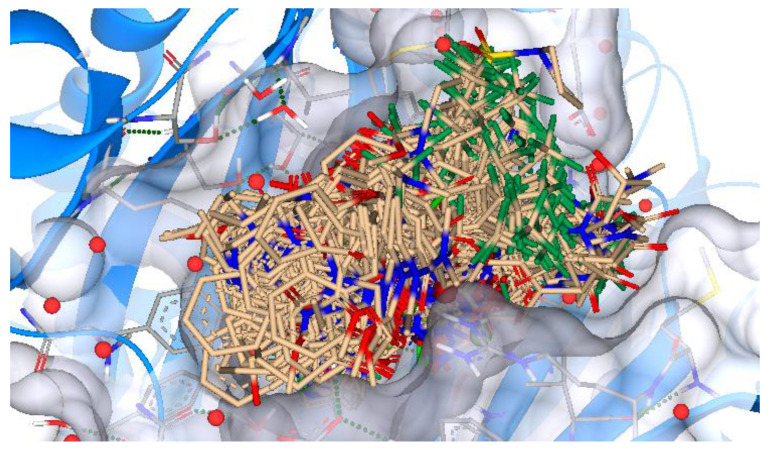
Molecular docking results. The image visualizes all the 540 poses generated from the docking of 54 compounds that were generated by Inspirator mode of SeeSAR based on the nitisinone nucleus.

The nitisinone molecule was shifted to Inspirator mode, which modifies the compounds using ReCore. Here, the groups of atoms, having unfavorable HYDE energy were replaced with appropriate fragments that were generated from the fragment library by ReCore. This process resulted in 54 new compounds that were moved to docking mode functionality in SeeSAR, where 540 poses were generated (10 for each compound), as represented in Figure 6.

### 2.5. Selection of Best Hits

**Figure 7:**
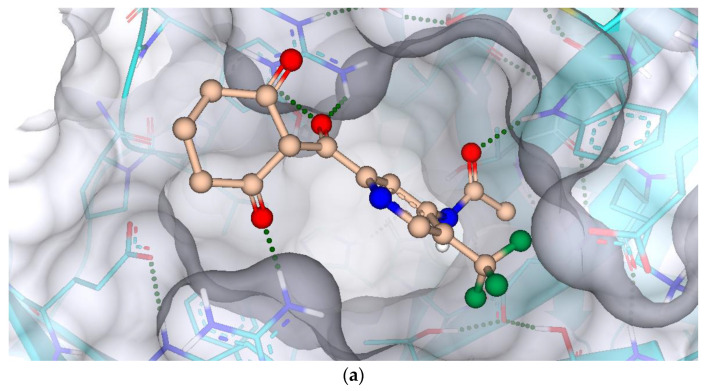

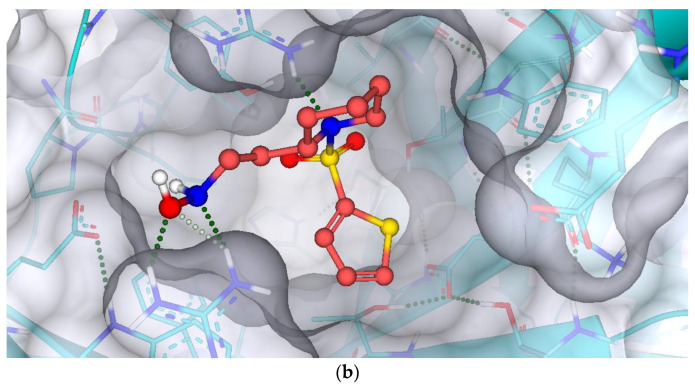
Docking complexes of compound **c** (**a**) and compound **f** (**b**) on the basis of highest affinities, minimum binding energies, no or minimum torsions, and the absence of any intra- and inter-molecular clashes.

**Figure 8:**
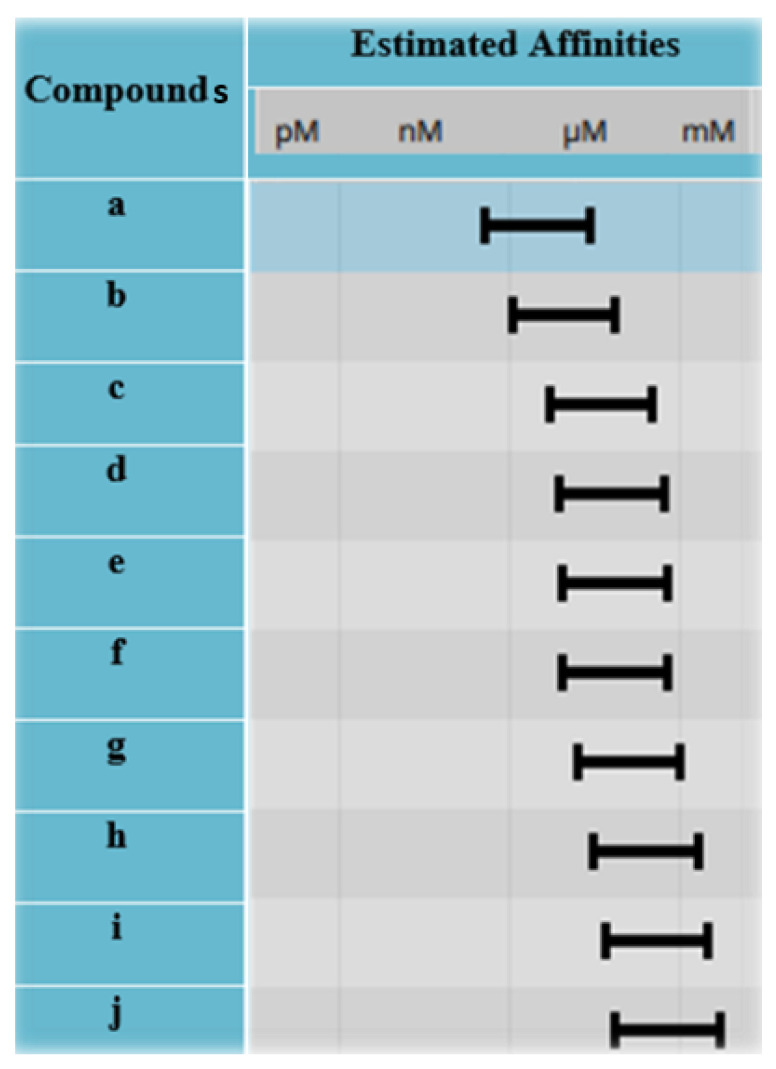
Estimated affinities of best hits. According to the estimated affinities, compound **a** shows the highest estimated affinity (via SeeSAR) than others.

Selection of hits and their optimization is a crucial step in the drug discovery process as it determines the success of the subsequent stages of drug development [41]. The use of bioinformatics tools, such as SeeSAR BioSolveIT, has greatly enhanced the hit selection process, enabling researchers to effectively identify the most promising compounds for further testing. Therefore, all the 540 poses were exported from docking mode to analyzer mode where their estimated affinities, torsions, clashes, and optibrium properties were calculated. A total of 10 compounds were obtained after screening that have the highest binding affinities, least torsions, no clashes, and low molecular weight, as shown in Table 2. They were named in alphabetical order from **a** to **j**. Among them, compound **h** is nitisinone. The docked complexes of compounds **c** and **f** with their best hits are shown in Figure 7, while the best hits for compounds **a**, **b**, **d**, **e**, and **g**–**h** are given in Supplementary Materials Figures S1–S8. The binding affinities of all 10 compounds (**a**–**j**) are shown in Figure 8.

### 2.6. ADME Analysis

**Figure 9:**
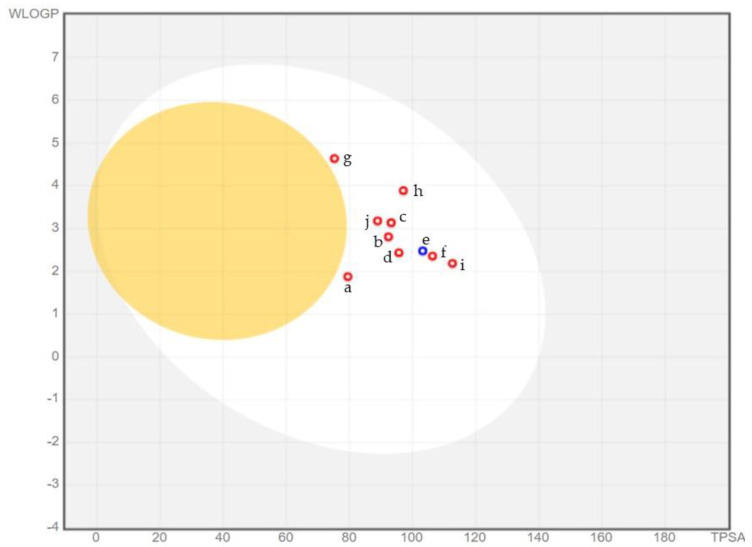
According to the boiled egg, all the selected best hits do not cross the blood–brain barrier (BBB) and have high gastrointestinal permeability.

The ADME analysis was performed by SwissADME. According to ADME properties, the selected hits have molecular weight less than 500 g/mol, hydrogen bond acceptors were less than 10, molar refractivity was between 57.75 and 76.07, and topological polar surface area (TPSA) ranged from 75.25 to 112.58 Å. Moreover, the consensus log P values were less than 1.99, whereas log S predicted that all the hits were soluble as elucidated in Table 3. Owing to pharmacokinetic properties, all the hits have high gastrointestinal absorption and were unable to cross the blood–brain barrier (BBB) depicting the safety of central nervous system, as shown in Figure 9 via the boiled egg. This study aims to synthesize drugs that will work outside the central nervous system (CNS) so drugs that will not cross the blood–brain barrier are preferred. The inhibition of cytochrome P450 (CYP) varies, while all obeyed Lipinski, Ghose, Veber, Egan, and Muegge drug-likeness rules with a 0.55 bioavailability score. Furthermore, no PAINS alerts have been observed and synthetic accessibility was from 2.06 to 3.76. All compounds showed lead likeness properties [42]. Compound **g, h**, and **j** were excluded because they have shown to inhibit the CYP, thus they can contribute to the toxicity when administered to the patient subsequent to further analysis. Compound **g** inhibits CYP1A2 and CYP2C19, while compound **h** inhibits CYP2C19. In addition, CYP1A2 is inhibited by compound **j**.

### 2.7. Protein-Ligand Interactions

Protein-ligand interactions perform a crucial role in many biological processes, including cell signaling, drug design, and protein–protein interactions. The identification and understanding of these interactions are essential for the development of new drugs and therapeutic strategies [43,44]. The docked complexes of hits were analyzed for interactions by using Discovery Studio. The data revealed that the compound **c** interacts with the active site of target protein by hydrogen bonding, halogen interaction, and pi-cation interaction. Three conventional hydrogen bonds are formed between the O9, and Trp25; O16, and Arg48; and O19, and Arg119 of the receptor. Similarly, the halogen interaction is formed between Glu81 of binding site and F4 of the ligand, whereas nitrogen containing heterocyclic aromatic ring of the ligand forms pi-anion bond with the Arg119 of the protein.

**Figure 10:**
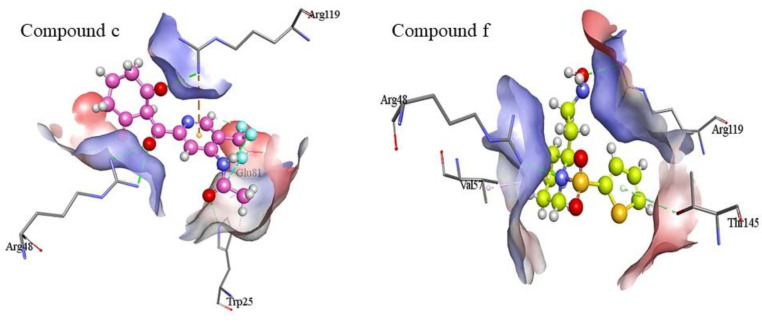
Visualization of ligand-protein interactions. Compounds **c** and **f** have demonstrated favorable interactions with the active site residues of the target protein. The dotted lines represent the interactions between residues and ligand. Arg48 and Arg119 in the active pocket of the receptor have common interactions with both the ligands.

Other than compound **c**, compound **f** also showed conventional hydrogen bonding with Arg48 and Arg119. These conventional hydrogen bonds are formed by the interaction of N4 and O9 with the amino groups (NH_2_) of Arg48 and Arg119, respectively. In addition, the pi-donor hydrogen bond is present between OG1 of Thr145 and the five cornered ring of the ligand. Contrarily, the ligand also form hydrophobic interaction with the ligand in the form of alkyl bond. This alkyl bond is formed between the Val57 of receptor and C11 of the ligand as shown in Figure 10. Compounds **a**, **b**, **d, e, g, h**, **I,** and **j** were excluded because of the unfavorable interactions, such as positive-positive interactions due to the presence of positively charged nitrogen in their structures.

### 2.8. Validation of Ligand Specificity

**Figure 11:**
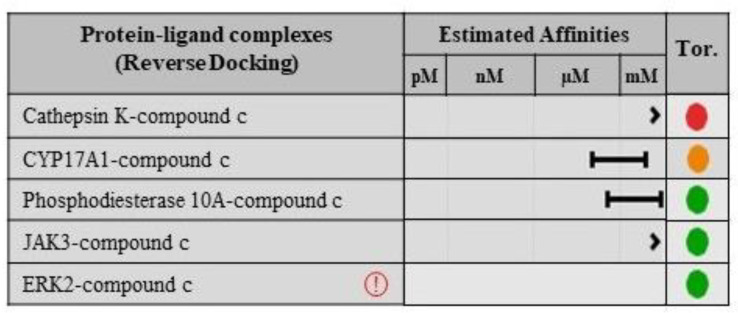
The estimated affinities of compound **c** with the predicted macromolecular targets obtained from molecular docking. The colored circles are depicting the torsion quality of the compound c upon docking with respective macromolecule where green color depicts no torsion, orange color shows bad torsion and red color represents worse torsion.

**Figure 12:**
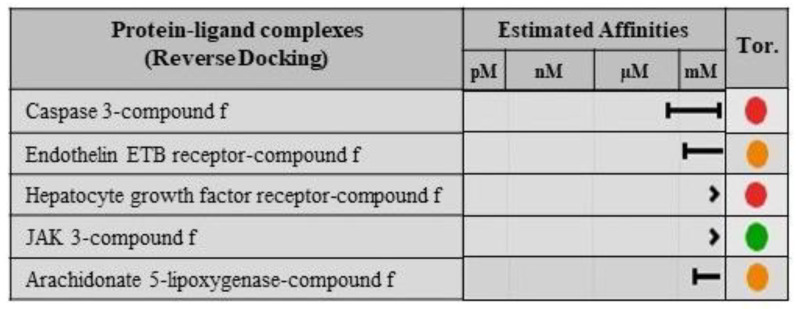
The estimated affinities of compound **f** with the predicted macromolecular targets obtained from molecular docking. The compound f torsion quality upon docking with respective macromolecule is depicted by colored circles, where green indicates no torsion, orange indicates bad torsion, and red represents worse torsion.

In order to validate the specificity of compounds **c** and **f**, reverse docking was performed. For this purpose, both compounds were docked against the druggable sites of macromolecular targets predicted by SwissTargetPrediction. Compound **c** does not show any appreciable affinity with all the predicted targets, which include cathepsin K, CYP17A1, phosphodiesterase 10A, JAK3, and ERK2, as shown in Figure 11. Likewise, compound **f** also exhibits least affinities with the macromolecular ligands predicted by SwissTargetPrediction, such as hepatocyte growth factor receptor, caspase 3, JAK 3, endothelin ETB receptor, and arachidonate 5-lipoxygenase as elucidated in Figure 12.

## 3. Methodology

### 3.1. Target Identification

The target for drug designing was selected from the literature via PubMed; moreover, the mutated protein in AKU was determined via the literature and the Kyoto Encyclopedia of Genes and Genomes (KEGG) pathway. KEGG is a database for metabolic pathways that consists of reference pathway databases to provide insight into the relative size and degree of overlap of these pathways [45]. A three-dimensional structure (3D) of the target was downloaded from Protein Data Bank (PDB) database-a sole repository of experimentally resolved 3D structures of large molecules across the world [46]. This selection of the protein was made by considering the resolution of protein (less than 2.5 Å), structure completion (greater than 90%), and availability of the ligand.

### 3.2. Binding Site Prediction

After loading the protein and selecting the co-crystalline ligand in the protein mode of SeeSAR12.1.0, the complex was moved to the binding site mode. Here, all the unoccupied spaces and residues at the active site were evaluated. Subsequent to the selection of an appropriate binding pocket, the number of residues at the selected active site were increased. The purpose was to enhance the number of interactive sites for ligands [47].

### 3.3. Ligand Evaluation

The structure of nitisinone (ligand) was downloaded from PubChem and evaluated in the analyzer mode of SeeSAR12.1.0 after performing its standard docking. The contribution of each atom in the overall binding affinity between ligand and target protein was investigated by HYdrogen bond and DEhydration energy (HYDE) scoring. The HYDE score is based on two parameters, namely, the hydrogen bond energy and the hydrophobic effect. In addition to score prediction, HYDE can also be visualized by a very intuitive coloring scheme that conveniently segregates the favorable and unfavorable contributions of atoms in the target-ligand complex [40,48].

### 3.4. ReCore and Molecular Docking

The unfavorable atoms were replaced with various fragments preserving conformational information and generating new compounds using ReCore [49]. ReCore is used for designing drugs based on fragments utilizing 3D fragment library known as “index” and is developed by BioSolveIT. It utilizes a vector-based scheme to generate 3D scaffolds altering the core elements of molecules within seconds [50,51]. Afterwards, these compounds were docked with the target protein using FlexX docking functionality in SeeSAR. This molecular docking depends on incremental construction algorithm, in which ligands are cleaved into fragments and each fragment is placed at multiple sites in the binding pocket [52].

### 3.5. Selection of Best Hits

Subsequent to docking, at least 10 poses were generated for each compound and their estimated affinities, torsions, clashes, and optibrium properties were analyzed in the Analyzer mode of SeeSAR. The estimated affinity ranges from millimolar to picomolar, whereas torsions and clashes can be visualized in the form of specific colors. The green color indicates the best results whereas orange and red colors represent the need to re-consider the bond angles and bond lengths between atoms [53]. Therefore, compounds with the best results were screened representing the best hits.

### 3.6. ADME Analysis

Absorption, distribution, metabolism, and excretion (ADME) properties of the hits were examined via SwissADME, an online tool that requires SMILES of compounds as input, and interprets results in the form of graphs, tables, and even in spreadsheet form. The interpretations include structure and bioavailability radar, physiochemical properties, lipophilicity, solubility, pharmacokinetics, drug-likeness, and medicinal chemistry of each input [54]. In addition, protein binding, CYP450 inhibition, and blood–brain barrier permeability are also predicted. This information can be used to assess the likelihood of a drug candidate being successful in clinical trials, and its potential for efficacy and safety [55].

### 3.7. Protein-Ligand Interactions

**Figure 13:**
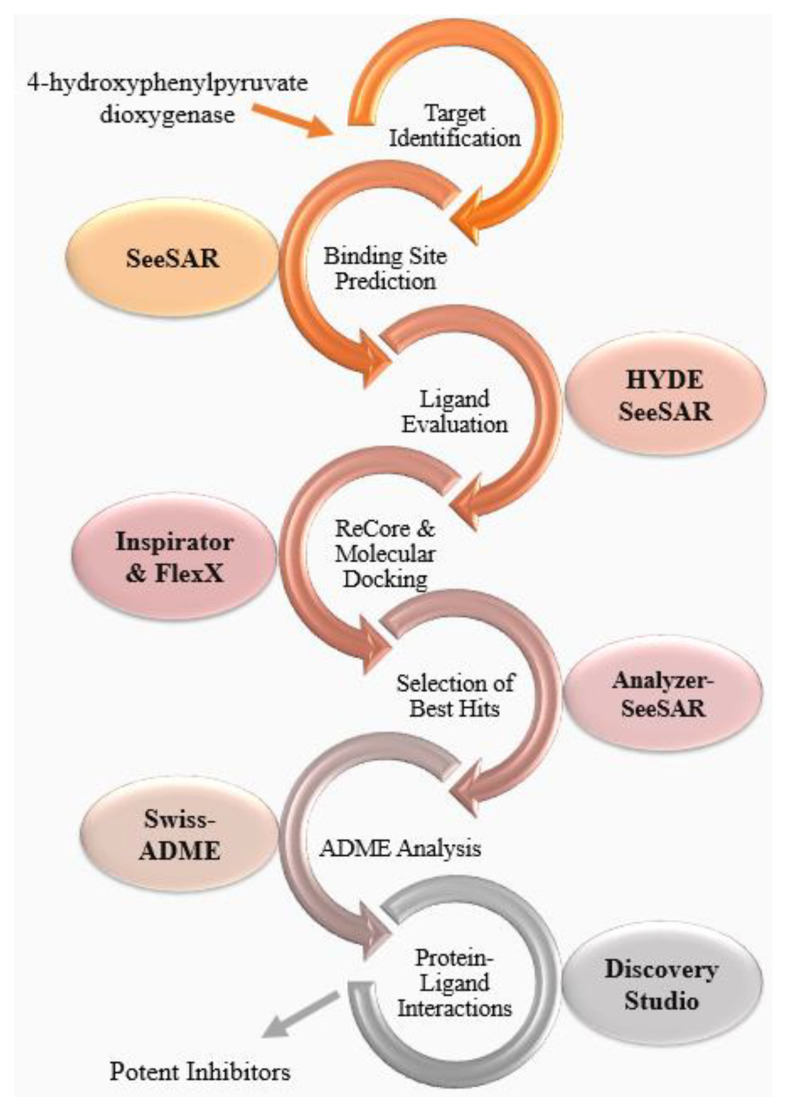
Overview of methodology. This flowchart describes the steps and the software used to perform each step.

Further analysis and examination of docked complexes were carried out through Discovery Studio 2021 molecular visualization software, which represents the 2D interactions between ligand atoms and specific amino acids in the active site [56]. These interactions include hydrogen bonds, hydrophobic interactions, van der Waal forces, and electrostatic interactions represented by specifically colored dotted lines [57]. An overview of the methods used in this study is shown in Figure 13.

## 4. Conclusions

Alkaptonuria, a congenital disorder of tyrosine metabolism, leads to a build-up of a substance called homogentisic acid (HGA) in the body, which can lead to a range of health issues. The disease is caused by mutations in the HGD gene and is passed down from parent to child in an autosomal recessive pattern. Symptoms usually begin to appear in adulthood, and can include joint pain and stiffness, arthritis, and heart valve damage. There is currently no cure for alkaptonuria, but treatments are available to manage its symptoms and prevent complications. Therefore, novel compounds based on the structure of nitisinone were produced by fragment replacement feature of ReCore. Upon docking of these compounds, the hits were screened and analyzed by SwissADME for potent inhibitors. Compound **h** is nitisinone, which not only inhibits CYP, but also exhibits unfavorable positive-positive interactions with the protein binding site. Compounds **c** and **f** showed optimum druggable properties against 4-hydroxyphenylpyruvate dioxygenase. These potent compounds do not inhibit cytochromes and have high gastrointestinal absorption. Moreover, these compounds can be synthesized and could exhibit lead-likeness with no PAINS alerts. The selected potent inhibitors showed better estimated affinities than nitisinone. The acquired in silico results showed promising compounds that could further be validated through experimental work and serve as a potential treatment for the very rare inherited disorder, alkaptonuria.

## Figures and Tables

**Table 1:** Unoccupied binding pockets predicted via binding mode in SeeSAR.

Pocket ID		Number of Residues	DoGSiteScore	Number of Donors	Number of Acceptors	Hydrophobicity	Solvent Accessible Surface (Å^2^)	Total Volume of the Pocket (Å^3^)
	1	23	0.42	11	14	0.71	283.32	538.27
	2	24	0.39	11	8	0.76	267.12	247.75
	3	17	0.34	4	12	0.77	194.40	164.16
	4	25	0.31	18	14	0.67	392.04	477.36
	5	16	0.31	5	9	0.76	158.76	170.86
	6	18	0.28	11	9	0.73	200.16	237.60
	7	9	0.16	3	6	0.74	86.04	112.10
	8	27	0.16	17	19	0.67	293.76	266.33
	9	12	0.12	6	8	0.69	129.60	192.89
	10	17	0.11	6	9	0.67	171.36	239.11

The different colors in the pocket ID are depicting the unoccupied binding sites that can be easily visualized in the Figure 3.

**Table 2:** Structures of hits **a**–**j**.

Compounds	Structures	Compounds	Structures
**a**	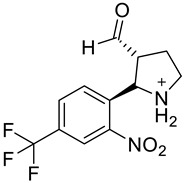	**b**	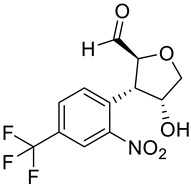
**c**	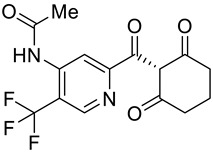	**d**	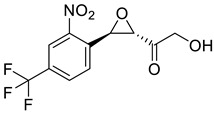
**e**	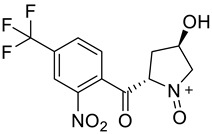	**f**	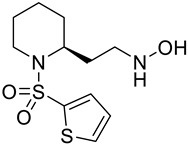
**g**	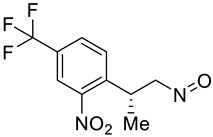	**h**	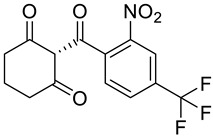
**i**	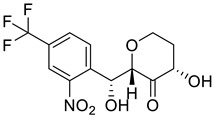	**j**	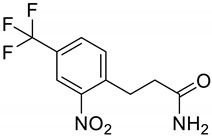

**Table 3:** ADME analysis of the screened hits after molecular docking via SeeSAR.

Compounds	a	b	c	d	e	f	g	h	i	j
Formula	C_12_H_12_F_3_N_2_O_3_+	C_12_H_10_F_3_NO_5_	C_15_H_13_F_3_N_2_O_4_	C_11_H_8_F_3_NO_5_	C_12_H_10_F_3_N_2_O_5_+	C_11_H_18_N_2_O_3_S_2_	C_10_H_9_F_3_N_2_O_3_	C_14_H_10_F_3_NO_5_	C_13_H_12_F_3_NO_6_	C_10_H_9_F_3_N_2_O_3_
Molecular weight	289.23 g/mol	305.21 g/mol	342.27 g/mol	291.18 g/mol	319.21 g/mol	290.40 g/mol	262.19 g/mol	329.23 g/mol	335.23 g/mol	262.19 g/mol
Heavy atoms	20	21	24	20	22	18	18	23	23	18
Aromatic heavy atoms	6	6	6	6	6	5	6	6	6	6
Fraction Csp^3^	0.42	0.42	0.40	0.36	0.42	0.64	0.40	0.36	0.46	0.30
Rotatable bonds	4	4	5	5	4	5	5	4	4	5
H-bond acceptors	6	8	8	8	8	5	7	8	9	6
H-bond donors	1	1	1	1	1	2	0	0	2	1
Molar refractivity	70.23	64.79	76.07	59.99	73.05	74.48	59.58	72.78	70.76	57.75
TPSA (Å^2^)	79.50	92.35	93.20	95.65	103.20	106.26	75.25	97.03	112.58	88.91
Consensus Log *P*_o/w_	1.13	1.20	1.76	1.14	−0.17	1.54	2.56	1.99	0.99	1.62
Class	Soluble	Soluble	Soluble	Soluble	Soluble	Soluble	Soluble	Soluble	Soluble	Soluble
GI absorption	High	High	High	High	High	High	High	High	High	High
BBB permeant	No	No	No	No	No	No	No	No	No	No
P-gp substrate	No	No	No	No	Yes	No	No	No	No	No
CYP1A2 inhibitor	No	No	No	No	No	No	Yes	No	No	Yes
CYP2C19 inhibitor	No	No	No	No	No	No	Yes	Yes	No	No
CYP2C9 inhibitor	No	No	No	No	No	No	No	No	No	No
CYP2D6 inhibitor	No	No	No	No	No	No	No	No	No	No
CYP3A4 inhibitor	No	No	No	No	No	No	No	No	No	No
Log *K*_p_ (skin permetion)	−6.90 cm/s	−7.27 cm/s	−7.73 cm/s	−7.20 cm/s	−7.35 cm/s	−7.06 cm/s	−6.07 cm/s	−6.67 cm/s	−7.22 cm/s	−6.66 cm/s
Lipinski	Yes; 0 violation	Yes; 0 violation	Yes; 0 violation	Yes; 0 violation	Yes; 0 violation	Yes; 0 violation	Yes; 0 violation	Yes; 0 violation	Yes; 0 violation	Yes; 0 violation
Ghose	Yes	Yes	Yes	Yes	Yes	Yes	Yes	Yes	Yes	Yes
Veber	Yes	Yes	Yes	Yes	Yes	Yes	Yes	Yes	Yes	Yes
Egan	Yes	Yes	Yes	Yes	Yes	Yes	Yes	Yes	Yes	Yes
Muegge	Yes	Yes	Yes	Yes	Yes	Yes	Yes	Yes	Yes	Yes
Bioavailability score	0.55	0.55	0.55	0.55	0.55	0.55	0.55	0.55	0.55	0.55
PAINS	0 alert	0 alert	0 alert	0 alert	0 alert	0 alert	0 alert	0 alert	0 alert	0 alert
Lead-likeness	Yes	Yes	Yes	Yes	Yes	Yes	Yes	Yes	Yes	Yes
Synthetic accessibility	3.07	3.47	2.63	3.27	3.23	3.52	2.76	2.46	3.76	2.06

## Data Availability

Not applicable.

## Supplementary Materials

The following supporting information can be downloaded at: https://www.mdpi.com/article/10.3390/molecules28062623/s1, Figures S1–S8. The docked complexes of compounds **a**, **b**, **d**, **e**, and **g**–**h** with their best hits.
