# Supplementary material for: Identification of Potential Inhibitors for the Treatment of Alkaptonuria Using an Integrated In Silico Computational Strategy
Source: Molecules. 2023 Mar 14;28(6):2623. doi: 10.3390/molecules28062623 (PMC10058836; doi:10.3390/molecules28062623)
Supplement: Supplementary file 1 [file molecules-28-02623-s001.zip › molecules-2235394-supplementary.pdf]

Supporting Information

Article

Identification of Potential Inhibitors for the Treatment of Alkaptonuria Using an Integrated In Silico Computational Strategy

Sumera Zaib [1,*], Nehal Rana [1], Nadia Hussain [2,3], Hanan A. Ogaly [4,5] Ayed A. Dera [6] and Imtiaz Khan [7,*]

1 Department of Basic and Applied Chemistry, Faculty of Science and Technology, University of Central Punjab, Lahore-54590, Pakistan
2 Department of Pharmaceutical Sciences, College of Pharmacy, Al Ain University, Al Ain, UAE
3 AAU Health and Biomedical Research Center, Al Ain University, Abu Dhabi, UAE
4 Chemistry Department, College of Science, King Khalid University, Abha 61421, Saudi Arabia
5 Biochemistry and Molecular Biology Department, Faculty of Veterinary Medicine, Cairo University, Giza 12211, Egypt
6 Department of Clinical Laboratory Sciences, College of Applied Medical Sciences, King Khalid University, Abha, Saudi Arabia
7 Manchester Institute of Biotechnology, The University of Manchester, 131 Princess Street, Manchester M1 7DN, United Kingdom
* Correspondence: sumera.zaib@ucp.edu.pk (S.Z.); kimtiaz@hotmail.co.uk (I.K.)

Supporting Information

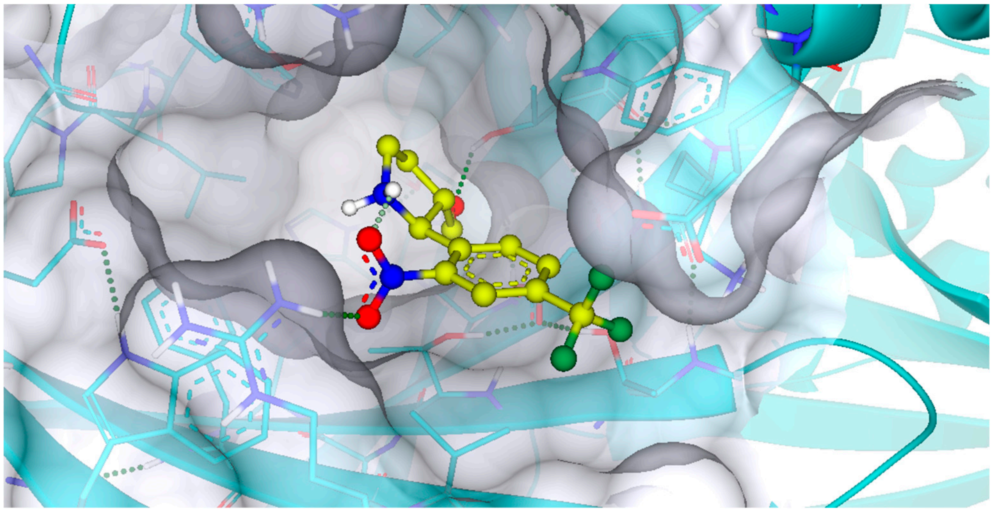

Figure S1. Docking complex of best hit of compound **a** on the basis of highest affinity, minimum binding energy, no or minimum torsion as well as the absence of any intra- and inter-molecular clashes.

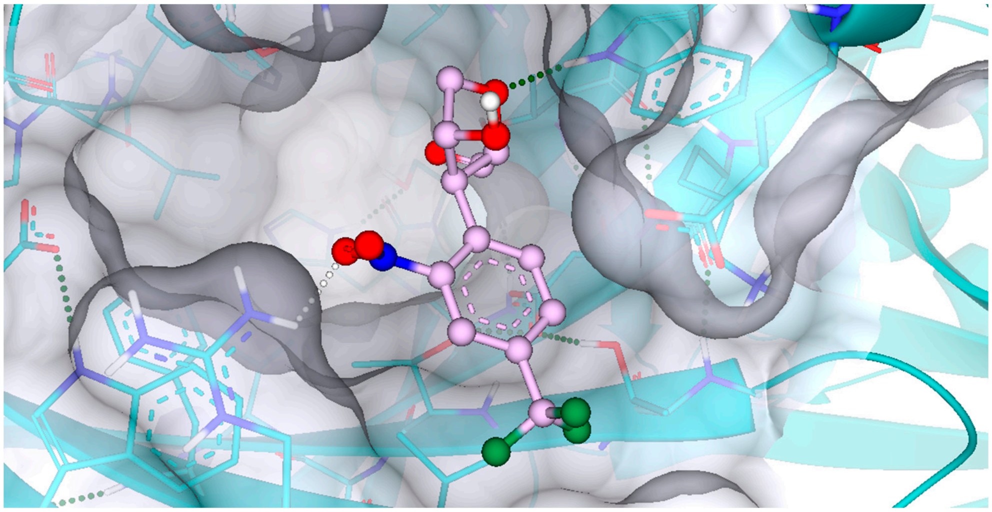

Figure S2. Docking complex of best hit of compound **b** on the basis of highest affinity, minimum binding energy, no or minimum torsion as well as the absence of any intra- and inter-molecular clashes.

Supporting Information

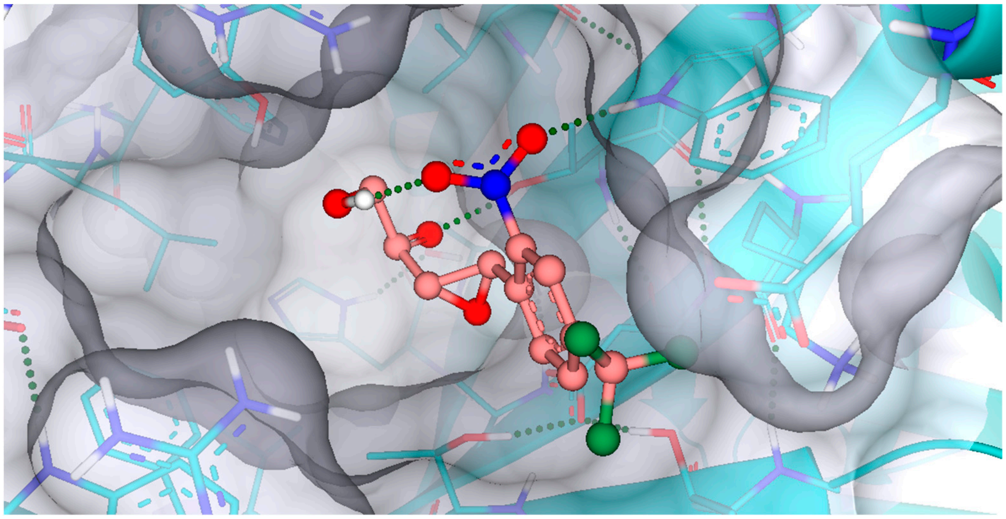

Figure S3. Docking complex of best hit of compound **d** on the basis of highest affinity, minimum binding energy, no or minimum torsion as well as the absence of any intra- and inter-molecular clashes.

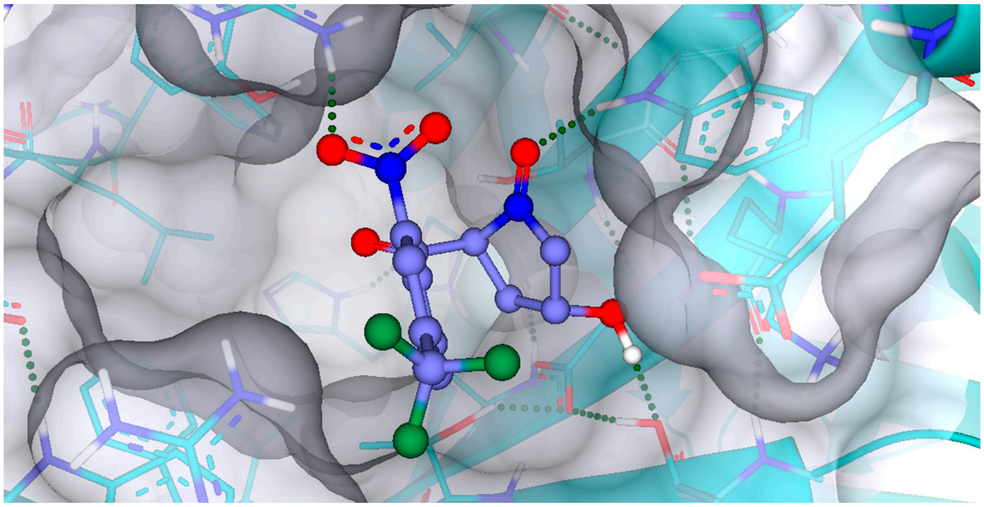

Figure S4. Docking complex of best hit of compound **e** on the basis of highest affinity, minimum binding energy, no or minimum torsion as well as the absence of any intra- and inter-molecular clashes.

Supporting Information

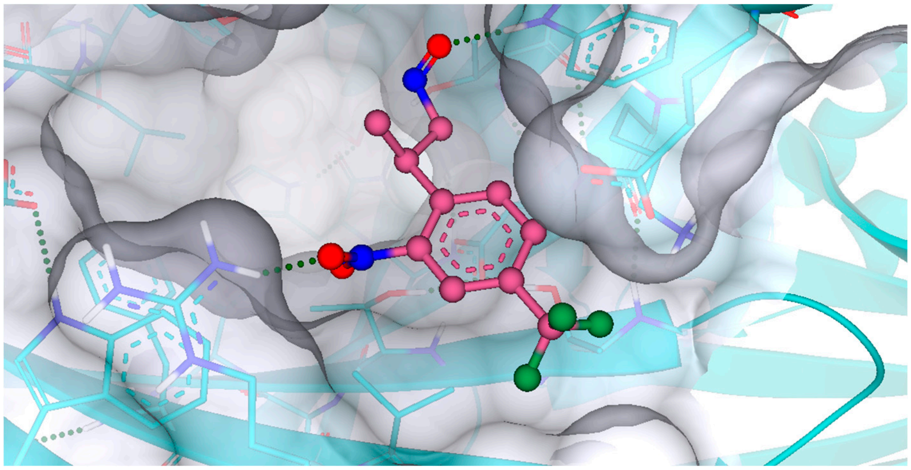

Figure S5. Docking complex of best hit of compound **g** on the basis of highest affinity, minimum binding energy, no or minimum torsion as well as the absence of any intra- and inter-molecular clashes.

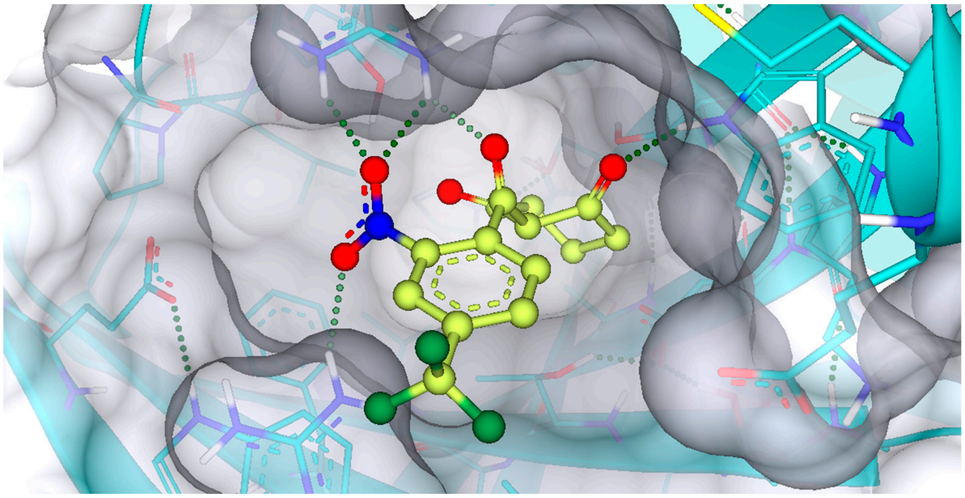

Figure S6. Docking complex of best hit of compound **h** on the basis of highest affinity, minimum binding energy, no or minimum torsion as well as the absence of any intra- and inter-molecular clashes.

Supporting Information

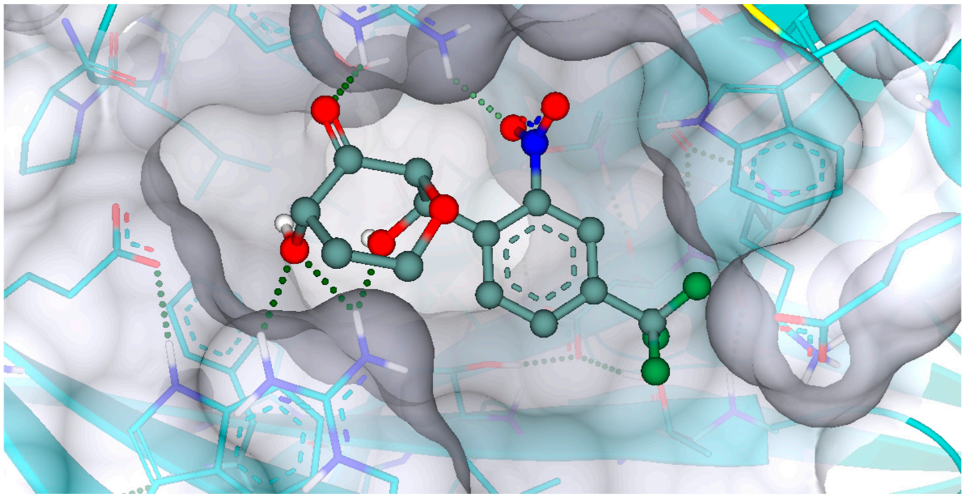

Figure S7. Docking complex of best hit of compound **i** on the basis of highest affinity, minimum binding energy, no or minimum torsion as well as the absence of any intra- and inter-molecular clashes.

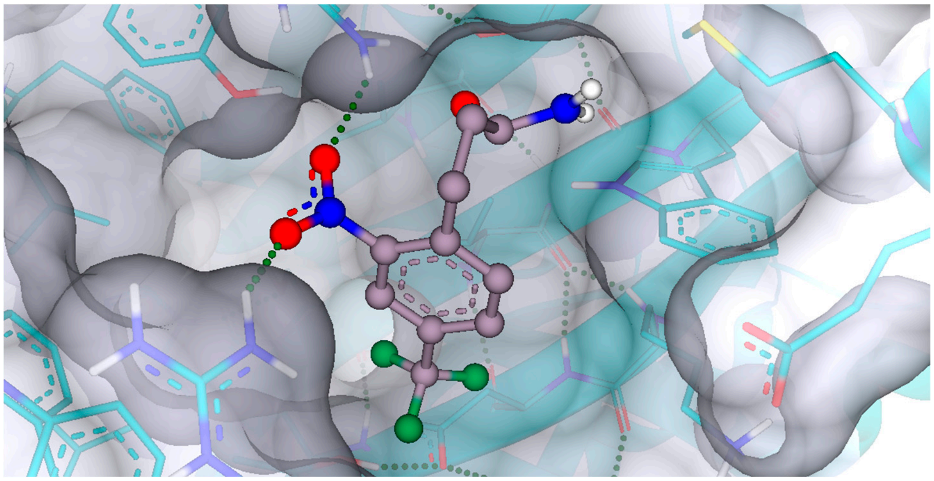

Figure S8. Docking complex of best hit of compound **j** on the basis of highest affinity, minimum binding energy, no or minimum torsion as well as the absence of any intra- and inter-molecular clashes.
